# Semi-Quantitative Versus Visual Analysis of Adenosine Perfusion Magnetic Resonance Imaging in Intermediate-Grade Coronary Artery Stenosis Using Fractional Flow Reserve as the Reference: A Pilot Study

**DOI:** 10.5334/jbsr.2675

**Published:** 2022-06-24

**Authors:** Olivier Ghekiere, Jean-Nicolas Dacher, Willem Dewilde, Wilfired Cools, Paul Dendale, Alain Nchimi

**Affiliations:** 1 Department of Radiology, Jessa Ziekenhuis, Stadsomvaart 11, B-3500 Hasselt, BE; 2 Department of Radiology, Centre Hospitalier Chrétien (CHC, Rue de Hesbaye, 75, B-4000 Liège, BE; 3 Faculty of Medicine and Life Sciences, Biomed and Reval, Hasselt University, Agoralaan, Building A and C, B-3500 Hasselt, BE; 4 Department of Radiology, CHU de Rouen, Normandie UNIV - UNIROUEN, Inserm U1096, F-76000 Rouen, FR; 5 Department of Cardiology, Imelda Hospital, Imeldalaan 9, B-2820 Bonheiden, BE; 6 Interfaculty Center data processing and Statistics, Vrije Universiteit Brussel (VUB) Pleinstraat 2, B-1050 Brussels, BE; 7 Heart Center Hasselt, Jessa Ziekenhuis Stadsomvaart 11, B-3500 Hasselt, BE; 8 Centre Hospitalier de Luxembourg, 4, Rue Ernest Barble L-1120 Luxembourg, LU

**Keywords:** Coronary artery stenosis, perfusion magnetic resonance imaging, adenosine, fractional flow reserve, semi-quantitative analysis

## Abstract

**Background:**

To evaluate the diagnostic accuracy of semi-quantitative adenosine perfusion magnetic resonance imaging (MRI) to determine fractional flow reserve (FFR) ≤ 0.80 intermediate-grade coronary stenoses as compared to visual analysis.

**Methods:**

Forty-six patients (mean age 61 ± 9 years; 33 males) with 49 intermediate-grade stenoses (59 ± 7.6%; range, 42–70% minimal diameter reduction) underwent adenosine perfusion MRI and FFR measurement within four months in this retrospective study. MRI was visually assessed by two experienced readers twice with one-year interval, the second time with the knowledge of the diseased artery. The stress subendocardial myocardial enhancement maximal upslope was evaluated distal to the coronary stenosis (=RISK) and divided by the same value in remote myocardium supplied by normal arteries (=REMOTE) to obtain the relative myocardial perfusion index (RMPI).

**Results:**

The average FFR value was 0.84 ± 0.09 and 15/49(31%) intermediate-grade stenoses were FFR ≤ 0.80. The kappa-values for interobserver agreement assessing inducible perfusion defects on visual readings was 0.20 on the first reading and increased to 0.62 with the knowledge of the stenosis location. Consensus readings had a diagnostic accuracy of 82%(40/49) in identifying FFR ≤ 0.80 stenoses on both blinded and unblinded readings with regards to the knowledge of the stenosis location. Meanwhile, stress subendocardial RMPI had higher accuracy (43/49[88%]) than visual readings to predict FFR ≤ 0.80 stenoses, using a cutoff value of 0.84.

**Conclusion:**

By assessing perfusion changes in remote myocardium, semi-quantitative MRI analysis using stress subendocardial RMPI can provide an equal or more accurate alternative to visual analysis in identifying FFR ≤ 0.80 intermediate-grade stenoses. Larger cohorts of patients are required to validate this approach.

## Background

Coronary computed tomography angiography (CCTA) and catheter coronary angiography poorly predict flow limitation, especially for stenoses in the intermediate-grade range (i.e. 40%–70% diameter reduction) [1,2] that may represent up to 42% of coronary stenoses [3]. Additional functional assessment is often required to guide therapeutic management as approximately only one-third of patients with intermediate-grade stenoses suffer from ischemia and would benefit from revascularization [4,5].

Invasive fractional flow reserve (FFR) measurement is the standard of reference for the functional significance (ischemia) of coronary stenoses. However, its use as a first step in intermediate-grade lesions is prevented by its invasiveness, the use of ionizing radiation and the costs of pressure wires [6,7]. Moreover, the use of invasive FFR varies widely depending on the practice of interventional cardiologists.

While dobutamine stress echocardiography and single-photon emission computed tomography have moderate and good accuracy (72 and 87,9 %) for identifying FFR-altered (i.e. FFR ≤ 0.8) intermediate-grade stenoses, no study has specifically addressed this subgroup of stenoses using stress perfusion magnetic resonance imaging (MRI) [8,9]. Though it has a higher accuracy for the detection of myocardial ischemia as compared to other non-invasive imaging modalities [10], visual analysis of adenosine perfusion MRI in daily clinical practice may be misleading compared to FFR.

The concept of FFR has been validated as a relative flow reserve such as the ratio of hyperemic flow pressure in a stenotic coronary artery to the hyperemic flow pressure in a normal coronary artery, so that the influence of microvascular resistance and collateral flow on the FFR value are minimized [11,12]. The relative myocardial perfusion index (RMPI) on stress perfusion MRI emulates the FFR concept by dividing the ratio of the maximal enhancement upslope distal to a coronary artery stenosis to that of a normally perfused area. As a result, RMPI is significantly higher correlated with FFR than perfusion analysis distal to the coronary artery stenosis [13].

Accordingly, the aim of this study was to evaluate the diagnostic accuracy of stress RMPI to determine FFR ≤ 0.80 intermediate-grade coronary stenoses as compared to visual analysis.

## Material and Methods

### Patients and study protocol

This study protocol was approved by the local institutional ethics committee, and patients provided written informed consent. Between 2010 and 2013, consecutive patients with an intermediate-grade stenosis on CCTA involving one or two major epicardial coronary vessels > 1.5 mm in diameter were eligible for a study requiring both catheter coronary angiography with FFR measurements and adenosine perfusion MRI within four months as previously reported. MRI examinations were performed on a 1.5T MR scanner (Avanto, Siemens Healthineers), as previously reported and described in the supplemental materials [13]. In short, the examination consisted in performing stress and resting perfusion on dynamic contrast-enhancement imaging (each using 0.1 mmol/kg Gadodiamide, Omniscan^®^), and late-gadolinium enhancement (LGE) in the same three short-axis positions (see the supplementary materials for more details). These acquired data were analyzed retrospectively as follows: visually twice with one-year interval, the second reading with knowledge of the diseased coronary artery; and semi-quantitatively accounting for perfusion changes in remote myocardium in predicting the FFR value.

### MRI analysis

#### Visual analysis

Two readers (AN, JND) with more than ten-years of experience in cardiac MRI, blinded to patient’s characteristics, history and coronary angiography and FFR findings, performed twice an individual visual analysis of perfusion MRI, using dedicated software (Syngo Via^®^, Siemens Healthineers). All reading discordances were noted for interobserver agreement and solved by consensus. First, splenic switch-off was qualitatively assessed to evaluate the appropriateness of the vasodilatation response after adenosine administration. An absent splenic switch-off represents a failure of the stressor to induce maximal vasodilatation at risk for false-negative ischemia testing [14]. Myocardial ischemia was defined as stress-induced myocardial perfusion defect in the absence of LGE in the same segment, as previously reported [3]. The readers had no common training before the study and had freedom to adjust the display window level and width. Twelve months after the first readings, a second round of consensus visual analysis was performed by both readers who then were provided with full knowledge of the coronary stenosis location, but still blinded to the FFR data.

An expert in cardiac MRI, blinded to the FFR data, adjudicated the correlation between coronary stenosis and myocardial perfusion defects using dedicated software (MOCO, Syngo Via30^®^, cardiac Engine-perfusion module, Siemens Healthineers). He reviewed all imaging files (CCTA and perfusion MRI) to determine the correlation between each coronary stenosis and the myocardial segments using both the 17-segment model of the American Heart Association and the coronary dominancy.

#### Semi-quantitative analysis

**Figure 1:**
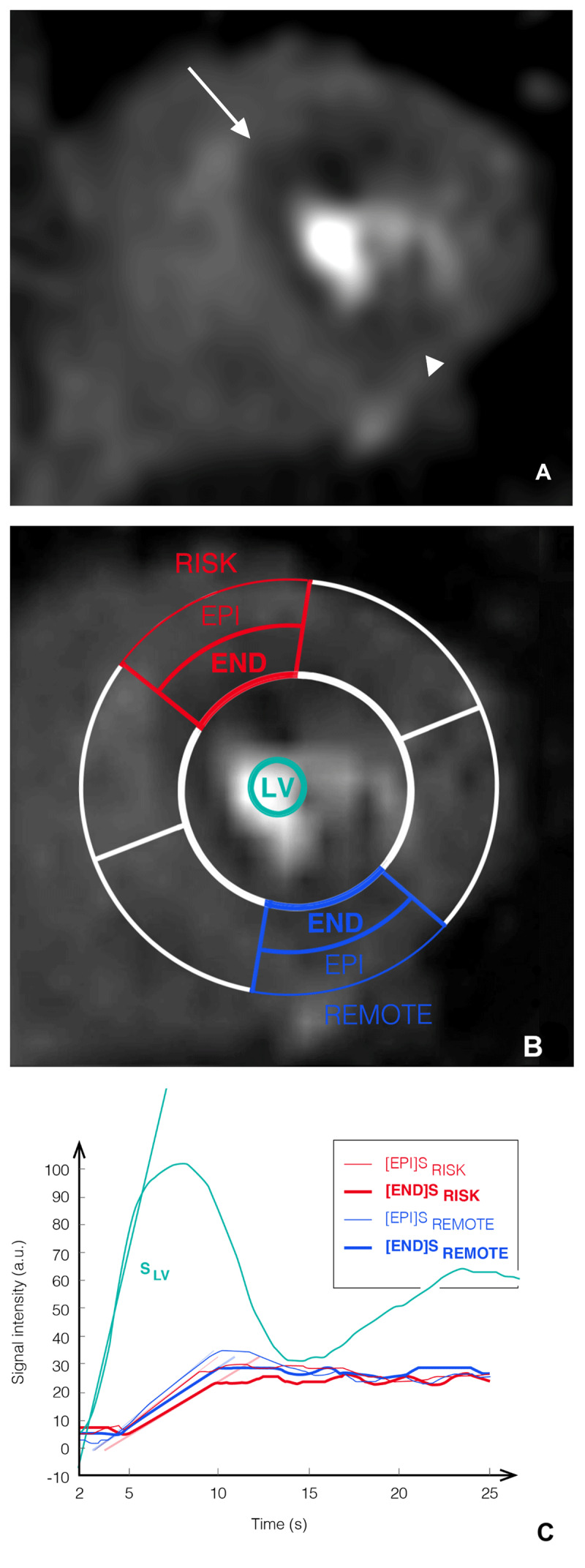
Semi-quantitative myocardial perfusion magnetic resonance imaging analysis. Detailed steps for semi-quantitative perfusion magnetic resonance imaging (MRI) analysis in a 69-year-old patient with intermediate-grade stenosis of the mid portion of the left anterior descending artery on both coronary computed tomography angiography and catheter coronary angiography (not shown). Peak myocardial enhancement on adenosine perfusion MRI showed a septal and anterior wall mid-left ventricular area of low signal intensity (white arrow, segment 7 in Figure 1A) in the area-at-risk (RISK), whereas the remote area (REMOTE) (arrowhead, segment 10 in Figure 1A) was homogenously enhanced. No abnormal enhancement was present on late-enhancement imaging (not shown). Equally divided subendocardial (bold lines, END) and subepicardial (thin lines, EPI) regions of interest are drawn in the RISK (red color) and REMOTE segments (blue color) after outlining the endocardial and epicardial borders of the myocardium during maximal hyperemia (Figure 1B). After extending these regions of interest to the whole frames, corresponding time-signal intensity curves and the maximal upslope value of the contrast enhancement were obtained (Figures 1C). RISK = myocardium beyond stenosis; REMOTE = remote myocardium; [END] = subendocardial; [EPI] = subepicardial; S= adenosine stress imaging.

As previously described [13], equally divided subendocardial (END) and subepicardial (EPI) regions of interest and time-signal intensity curves were obtained during adenosine stress in the myocardium distal to the stenosis (=RISK). When the RISK area involved more than one segment of the left ventricle representation, the myocardial segment with the greatest lateral and transmural extent of the perfusion defect was used for further measurements. Then, similar curves were obtained for a remote myocardial segment without a stenosis ≥40% diameter reduction on the supplying artery on QCA (=REMOTE) (Figure 1).

When no myocardial perfusion defect was visualized, the RISK segment was defined distal to the anatomic location of the coronary stenosis and the remaining steps were performed as when a perfusion defect could be visually detected. In patients with more than one intermediate-grade stenosis, each corresponding area of myocardial supply was assessed separately. If necessary, manual correction was made to adjust the region of interest placement.

The stress subendocardial RMPI (i.e.: RISK/REMOTE mean maximal enhancement upslopes) of each stenosis was assessed for the diagnosis of FFR ≤ 0.80 stenosis, as previously reported (Figure 1) [13].

### Statistical analysis

Statistical analyses were performed using R (version 3.2.3, with the model-based boosting package 2.6–0). Normally distributed continuous variables are expressed as mean +/- standard deviation (SD). Comparisons between continuous variables were performed using two-tailed Student t-tests, and comparisons of proportions were performed using χ² tests. Interobserver agreement for visual MRI analysis was calculated using Cohen’s κ test.

A regression model was fitted to determine the best cut-off value for stress subendocardial RMPI in predicting FFR ≤ 0.80. The diagnostic values were expressed as sensitivity, specificity, positive predictive value, negative predictive value, likelihood ratios and accuracy. The diagnostic accuracies were compared between visual readings and stress subendocardial RMPI for FFR ≤ 0.80, using binomial exact tests. P-values < 0.05 were considered to express a statistically significant difference.

## Results

### Patient and intermediate-grade coronary stenoses characteristics

**Figure 2:**
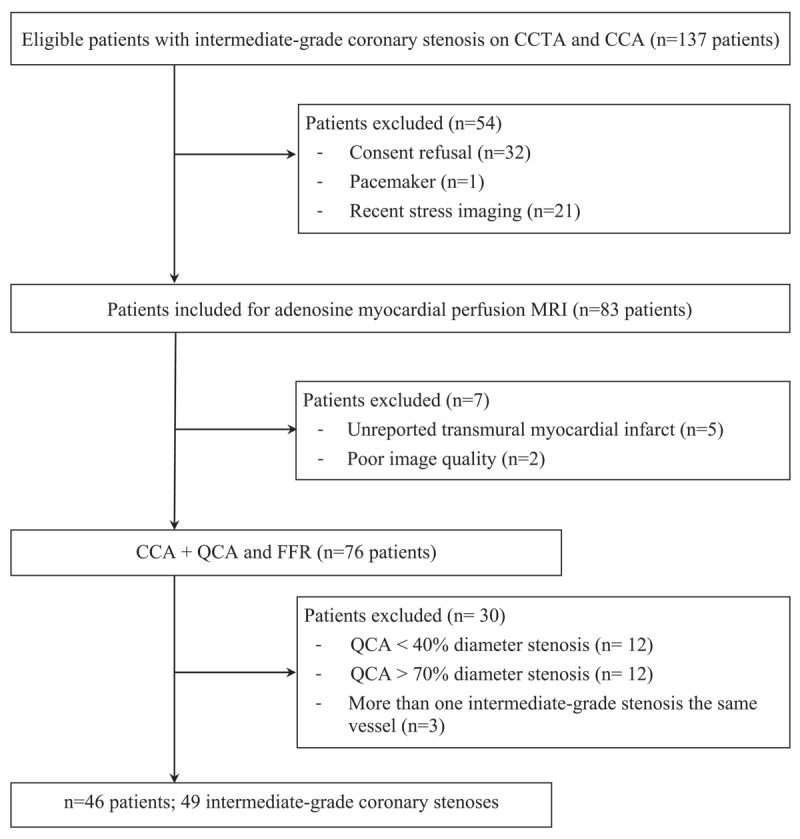
Study flowchart. CCTA = coronary computed tomography angiography; CCA = catheter coronary angiography; QCA = quantitative coronary angiography; FFR = fractional flow reserve; MRI = magnetic resonance imaging.

One hundred and thirty-seven patients fulfilled inclusion criteria, 54 were excluded because of consent refusal (n = 32), pacemaker (n = 1) and recent stress imaging (n = 21). Additionally, seven patients were excluded after MRI because of poor image quality (n = 2) and a segmental transmural myocardial infarct on LGE (n = 5); 30 patients were excluded after quantitative coronary angiography because actual stenosis was <40% (n = 12) or >70% (n = 12) minimal diameter reduction, because there were multiple intermediate-grade stenoses on the same artery (n = 3) (Figure 2), and >70% stenosis on another vessel (n = 3). In total, 46 patients were included (mean age 61 ± 9 years): 33 men (mean age 59 ± 9 years) and 13 women (mean age 67 ± 8 years). The demographics and cardiovascular risk factors are given in Table 1. Three of the 46 patients (6.5%) presented two intermediate-grade stenoses on distinct coronary arteries. Therefore, a total number of 49 intermediate-grade stenoses (59 ± 7.6% [range, 42–70%] minimal diameter reduction) were evaluated in this study (Table 2). The mean FFR value was 0.84 ± 0.09, with a range of 0.60 to 0.98; 31% (15/49) were ≤0.80.

**Table 1:** Patient demographics and cardiovascular risk factors.


PATIENT CHARACTERISTICS	NON ISCHEMIC (n = 31)	ISCHEMIC (n = 15)

Age (years)*	61 ± 9 [44–80]	62 ± 9 [48–80]

Ratio M/F	22/9	11/4

BMI (kg/m2)*	29 ± 5 [21–39]	27 ± 3 [24–35]

Resting heart rate (beats per minute)*	68 ± 13 [51–100]	67 ± 8 [54–81]

Family history of coronary disease	9 (29%)	3 (20%)

Personal history of coronary disease	3 (10%)	5 (33%)

Diabetes mellitus	10 (32%)	2 (13%)

Current tobacco smoker	10 (32%)	7 (47%)

Elevated blood lipid profile	23 (74%)	13 (87%)

Systemic hypertension	25 (80%)	8 (53%)

Agatston coronary calcium score**	225 [139–480]	465 [109–578]


* Mean ± standard deviation [range]. ** 4 males with coronary stenting excluded. M = male; F = female

**Table 2:** Segmental topography of 49 intermediate-grade coronary artery stenosis.


LOCATION	n (%)

Right coronary artery	12 (24.5)

Proximal segment	3 (6.1)

Mid segment	7 (14.3)

Distal segment	2 (4.1)

Left main trunk	1 (2)

Left anterior descending coronary artery	28 (57.2)

Proximal segment	14 (28.6)

Mid segment	14 (28.6)

Left circumflex coronary artery	7 (14.3)

Proximal segment	3 (6.1)

Mid segment	2 (4.1)

Distal segment	2 (4.1)

Marginal branch	1 (2)

First branch	1 (2)


### Visual analysis

Of the 46 examinations, the spleen was not visible in one case. Splenic switch-off on stress imaging was absent in two cases while present in the remaining patients (43/46; 93%). The diagnostic values of perfusion MRI for FFR ≤ 0.80 are given on Table 3, including readers’ visual consensus analyses. The first consensus reading yielded a sensitivity of 73% (11/15) and a specificity of 85% (29/34). With the knowledge of stenosis location on the second consensus reading, the diagnostic accuracy of the visual readings remained in the same range (sensitivity of 60 % (9/15) and a specificity of 91% (31/34). Interobserver agreement of the first visual reading session was 0.20 and increased to 0.62 with the knowledge of the stenosis location.

**Table 3:** Diagnostic values of visual and semi-quantitative analysis of adenosine perfusion MRI for FFR ≤ 0.80 intermediate-grade coronary artery stenoses.


INTERMEDIATE STENOSES (n = 49)	TP	TN	FP	FN	SENSITIVITY	SPECIFICITY	PPV	NPV	LR+	LR–	ACCURACY

**Visual consensus reading 1**	11	29	5	4	(11/15) 73%	(29/34) 85%	(11/16) 69%	(29/33) 87%	4.99	0.31	(40/49) 82%

**Visual consensus reading 2**	9	31	3	6	(9/15) 60%	(31/34) 91%	(9/12) 84%	(31/37) 84%	6.80	0.44	(40/49) 82%

**Relative myocardial perfusion**	12	31	3	3	(12/15) 80%	(31/34) 91%	(12/15) 80%	(31/34) 91%	4.56	0.11	(43/49) 88%


The proportions by which the percentages were calculated are given in parentheses. TP = true positive; TN = true negative; FP = false positive; FN = false negative; MRI = magnetic resonance imaging; FFR = fractional flow reserve; PPV = positive predictive value; NPV = negative predictive value; LR = likelihood ratio; relative myocardial perfusion = stress subendocardial relative myocardial perfusion index.

### Semi-quantitative analysis: RMPI

The values of myocardial time-signal intensity maximal upslope in RISK and REMOTE areas, normalized to the respective left-ventricle cavity enhancement upslope are summarized in Table 4, both in patients with ischemic and non-ischemic intermediate coronary artery stenosis. The stress subendocardial RMPI ranged between 0,57 and 1,39 (0,81 ± 0,17). Using the cutoff value of 0.84, the stress subendocardial RMPI had higher diagnostic accuracy (43/49, 88%) than the consensus readings to detect FFR ≤ 0.80 intermediate-grade coronary stenoses (Table 3).

**Table 4:** Semi-quantitative subendocardial stress enhancement parameters in RISK and REMOTE myocardium during adenosine perfusion in patients with ischemic and non-ischemic intermediate coronary artery stenosis (as defined by the 0.8 FFR cut-off value).


SUBENDOCARDIAL STRESS ENHANCEMENT PARAMETER	ISCHEMIC (FFR ≤0.80, n = 15)	NON-ISCHEMIC (FFR > 0.80, n = 31)

Mean maximal upslope in RISK myocardium*	0.16 ± 0.04 [0.11–0.24]	0.18 ± 0.05 [0.07–0.29]

Mean maximal upslope in REMOTE myocardium*	0.20 ± 0.04 [0.13–0.26]	0.18 ± 0.04 [0.09–0.28]

RMPI	0.79 ± 0.14 [0.57–1.05]	1.01 ± 0.13 [0.81–1.39]


Values are given as mean ± standard deviation [range]. * Maximal upslopes are normalized by the corresponding left ventricle cavity enhancement upslope; RISK = myocardium beyond stenosis; REMOTE = remote myocardium; RMPI = relative myocardial perfusion index; FFR = fractional flow reserve.

## Discussion

The current study heralds interesting findings regarding the workup for intermediate-grade stenoses via adenosine perfusion MRI: the use of a simple semi-quantitative index, RMPI, can be equal or better than experienced readers in identifying FFR ≤ 0.80 intermediate-grade stenoses.

In addition, interobserver agreement on visual analyses was highly variable between the first and second visual readings, likely owing to the freedom in image setting adjustment and artifact assessment. The agreement would have likely been higher, but less representative of the ‘real life’ if a pre-study training of the readers would have been organized [15]. These reading pitfalls suggest that the interpretation of myocardial signal abnormality depends on many more factors beyond the reader’s experience.

Consensus visual readings resulted in good diagnostic accuracy for FFR ≤ 0.80 stenosis, although lower than previous perfusion MRI studies [3,16,17]. This can be explained by the exclusive inclusion of intermediate-grade stenoses in our study, as the reported lower sensitivity of MRI in identifying FFR ≤ 0.80 lesions was also in line with a subanalysis of intermediate-grade stenoses from a larger series [18]. Only a few studies have specifically addressed coronary flow-limitation in intermediate-grade stenoses using other non-invasive techniques such as stress dobutamine MRI [19], dobutamine stress echocardiography [20], and single-photon emission tomography [9,21,22], with respective sensitivity and specificity ranges of 62%–95% and 69%–90%, all confirming the challenge posed by this range of stenoses.

The knowledge of the area-at-risk did not increase the accuracy of visual readings in our study. Indeed, the perfusion defects induced by intermediate-grade stenoses are likely to be shallower and less extended, thus more difficult to perceive and to distinguish from subendocardial dark-rim artefacts, than those caused by high-grade stenoses [23,24]. This implies that beyond encouraging consensus reading of perfusion MRI to mitigate the reader’s perception biases, diagnostic use of MRI as a gatekeeper to predict functional significance of intermediate-grade stenoses demands improvement. Actually, visual analysis assesses only perfusion defects beyond a coronary stenosis, and does not account for perfusion in normal perfusion areas, in contrast to the FFR value [11].

The reported stress subendocardial RMPI provides a simplified and useful semi-quantitative parameter for clinical practice [13], with a high diagnostic accuracy to determine FFR ≤ 0.80 intermediate-grade stenoses, in line with those of previous meta-analyses, including mainly semi-quantitative and quantitative MRI analyses [10]. This approach has also been reported with stress dynamic computed tomography perfusion showing better accuracy to identify flow-limiting stenoses than the myocardial blood flow in the area-at-risk [25]. Nevertheless, using RMPI three false-negative and three false-positive cases remained for the FFR ≤ 0.8 cutoff, owing to image artifacts and the existence of the so-called gray-zone of FFR values (0.75–0.80) [26]. With the advent of other techniques such as FFR computed by CCTA (FFR_CT_), it becomes questionable which of stress perfusion techniques and FFR_CT_ would represent the best strategy in patients with equivocal findings regarding disease severity on CCTA [27]. Compared to perfusion MRI, FFR_CT_ is probably better fitted to predict FFR and provides anatomical and functional evaluation of a given coronary stenosis, whereas the latter assesses myocardial perfusion that may be affected not only by epicardial coronary stenosis, but also by endothelial dysfunction and microvascular disease that are important determinants of outcomes in coronary artery disease. For this reason, perfusion MRI provides crucial information on top of that provided by FFR and FFR_CT_ [28,29].

Our study has certain limitations including the relatively low number of patients and FFR ≤ 0.80 stenosis. Second, the MRI studies were performed between 2010 and 2013 with an older generation equipment. Both visual and semi-quantitative analysis results could have been improved using a higher resolution adenosine perfusion MRI as it improves the detection of subendocardial ischemia [30]. Third, substantial amount of dropouts occurred after QCA to maintain stenoses within the intermediate-grade range and to control the possible bias related to the hemodynamic interactions between distinct coronary territories and that of successive stenosis. We nevertheless included a sufficient number of patients regarding the sample estimation for statistical significance, and approximately one-third of the intermediate-grade stenoses had an FFR ≤ 0.80, as reported in the literature. Larger cohorts of patients will be suitable to confirm and validate the results of our study. Finally, in spite of epicardial stenosis, several confounders may alter myocardial perfusion on MRI and, therefore, its diagnostic values in predicting flow-limitation as defined by the FFR value [31]. Systematic bias such as cardiac-phase variability of the myocardial perfusion was not taken into account [32,33], but can be ignored as long as a single-slice frame is evaluated, as it was done in this study. Patient-related confounders inherently limit the validity of our data to a population of individuals with similar cardiovascular risk factors for microvascular disease.

In conclusion, consensus reading should be encouraged in clinical practice for visual analysis of adenosine perfusion MRI. Semi-quantitative analysis using RMPI can provide an equal or better alternative to visual analysis in determining FFR ≤ 0.80 lesions among intermediate-grade stenoses detected on other diagnostic modalities such as coronary computed tomography angiography and catheter coronary angiography. Further studies with larger patient samples are needed to confirm its clinical value as a gatekeeper for invasive FFR.

## Date accessibility statement

The datasets used and/or analysed during the current study are available from the corresponding author on reasonable request.

## Additional File

The additional file for this article can be found as follows:

10.5334/jbsr.2675.s1 Supplemental Materials. MRI protocol.
