# Supplementary material for: Semi-Quantitative Versus Visual Analysis of Adenosine Perfusion Magnetic Resonance Imaging in Intermediate-Grade Coronary Artery Stenosis Using Fractional Flow Reserve as the Reference: A Pilot Study
Source: J Belg Soc Radiol. 2022 Jun 24;106(1):59. doi: 10.5334/jbsr.2675 (PMC9231575; doi:10.5334/jbsr.2675)
Supplement: Supplemental Materials. — MRI protocol. [file jbsr-106-1-2675-s1.pdf]

Supplemental materials

MRI protocol

MRI examinations were performed on a 1.5T MR (Avanto, Siemens Heathineers) equipped with a high gradient (40 mT/m/sec). A six-element phased-array cardiac coil (32 independent receiver channels) in combination with two elements of the spine array was used, allowing all image acquisition acceleration by a factor of 2 with parallel imaging.

Patients were asked to suspend the intake of beta-blockers and competitive antagonists of adenosine (caffeinated beverages) 24 h before the examination. On the day of examination, a 22-gauge catheter was inserted into an antecubital vein of both arms and connected to an automated pump pre-filled with adenosine (Adenocor®, Sanofi-Aventis) (Infusomat® FM) and to a double-chamber pump (Medtron, Medrad®) pre-filled with a 0.5 M gadolinium chelate contrast agent (Gadodiamide, Omniscan®) in one chamber and saline in the second chamber. Three MR-compatible torso leads were placed on the patients and connected to an MR-compatible monitoring system (Millenia 3500, Invivo). Patients were placed in the supine position under continuous heart rate and blood pressure monitoring.

After scouts and determination of the heart axes, perfusion MRI sequence was a prospective ECG-triggered 2D sequences of three 8-mm-thick slices in the short axis of the left ventricle. The central slice was placed at the level of the papillary muscles, and the remaining two were placed 8 mm on each side. Stress perfusion MRI was performed during maximal vasodilatation (i.e., 3 min after beginning the injection of 140 µmol/kg/min of adenosine using selective saturation-prepared T1-weighted steady-state free precession (SSFP) slices. We used symmetric echoes with TR = 2.2 ms, TE = 1.1 ms and flip angle = 50°; the resulting acquisition time per image

was 178 ms. The bandwidth was 1371 Hz/pix, the matrix size 192 x 115, resulting into an image resolution of 3.5x2.1x8.0 mm. Perfusion MRI acquisition started simultaneously with the injection of 0.1 mmol/kg of the contrast agent and a 30 mL saline flush; both at the rate of 4 mL/s. During a single breath hold, measurements of the 3 slices per heartbeat were acquired 50 times using a linear sampling of the k-space. The field-of-view (FOV) was adjusted to the subjects' size to avoid folding artifacts.

Approximately 10 min after the start of the intravenous injection of contrast agent, myocardial late-gadolinium enhancement (LGE) imaging was performed using breathhold phase-sensitive inversion recuperation (PSIR) sequences in identical slice thickness, planes and FOVs as perfusion MRI. PSIR images parameters were as follows: TR = 600 ms, TE = 3.4 ms, flip angle = 25°, TI = 260 ms, bandwidth = 130 Hz/pix, matrix size 256 x 192; resulting into an image resolution of 1.6x1.2x8.0 mm.

Resting myocardial perfusion images were subsequently acquired after PSIR imaging under the same technical conditions as the adenosine perfusion MRI.

Coronary angiography, quantitative coronary angiography and fractional flow reserve (FFR) measurements

All patients underwent coronary angiography by catheterization of a common femoral artery using a 7-French catheter. At least 2 orthogonal projections for all segments were performed after the injection of iodinated contrast medium (Hexabrix 320 mg/mL, Guerbet) and 100–200 lg intracoronary nitroglycerin infusion, using previously described standard procedures (1). Quantitative coronary angiography (QCA) analysis was subsequently performed with the Xcelera R3.1L1 system (Philips

Medical Systems) to confirm the intermediate-grade (40-70% diameter stenosis) of the stenosis detected on coronary CTA.

After placement of a 0.014 inch pressure sensor tipped coronary angioplasty guide wire across the intermediate-grade stenosis on QCA (FloWire_Doppler Guide Wire image Volcano Corporation), FFR was determined as the ratio between the absolute distal pressures recorded at rest and during maximal myocardial hyperemia, i.e. 30 to 60 s after intracoronary injection of 15 to 20 mg of papaverine (papaverine STEROP 100 mg/3 mL).

References

1. Tonino PA, De Bruyne B, Pijls NH, Siebert U, Ikeno F, van' t Veer M, et al. Fractional flow reserve versus angiography for guiding percutaneous coronary intervention. N Engl J Med. 2009;360(3):213-24. doi: 10.1056/NEJMoa0807611.
